# Determination of the Primary Molecular Target of 1,2,4-Triazole-Ciprofloxacin Hybrids

**DOI:** 10.3390/molecules20046254

**Published:** 2015-04-09

**Authors:** Tomasz Plech, Barbara Kaproń, Agata Paneth, Urszula Kosikowska, Anna Malm, Aleksandra Strzelczyk, Paweł Stączek, Łukasz Świątek, Barbara Rajtar, Małgorzata Polz-Dacewicz

**Affiliations:** 1 Department of Organic Chemistry, Faculty of Pharmacy, Medical University, Chodzki 4A, Lublin 20-093, Poland; E-Mails: baska_k@o2.pl (B.K.); agata.paneth@umlub.pl (A.P.); 2 Department of Pharmaceutical Microbiology, Faculty of Pharmacy, Medical University, Chodzki 1, Lublin 20-093, Poland; E-Mails: u.kosikowska@umlub.pl (U.K.); anna.malm@umlub.pl (A.M.); 3 Department of Genetics of Microorganisms, University of Lodz, Banacha 12/16, Lodz 90-237, Poland; E-Mails: ola.strzelczyk@wp.pl (A.S.); pstaczek@biol.uni.lodz.pl (P.S.); 4 Department of Virology, Faculty of Medicine, Medical University, Chodźki 1, Lublin 20-093, Poland; E-Mails: lukasz.swiatek@umlub.pl (Ł.Ś.); b.rajtar@umlub.pl (B.R.); malgorzata.polz-dacewicz@umlub.pl (M.P.-D.)

**Keywords:** fluoroquinolones, gyrase DNA, topoisomerase IV, topoisomerase inhibitors, MTT assay

## Abstract

We have synthesized and examined the antibacterial activity, toxicity and affinity towards bacterial type II topoisomerases of a series of 1,2,4-triazole-ciprofloxacin hybrids. A number of these compounds displayed enhanced activity against Gram-positive and Gram-negative bacteria when compared to ciprofloxacin. The toxic concentrations of the obtained derivatives, evaluated on HEK-293 cells using MTT assay, were much higher than concentrations required to produce antibacterial effect. Finally, the results of enzymatic studies showed that the analyzed compounds demonstrated other preferences as regards primary and secondary molecular targets than ciprofloxacin.

## 1. Introduction

A chance discovery of nalidixic acid, being a by-product in the synthesis of chloroquine, initiated the development of antibacterial drugs known as (fluoro)quinolones. Chemotherapeutics from the (fluoro)quinolones group characterize with a broad-spectrum of antibacterial effect, including Gram-positive and Gram-negative bacteria, mycobacteria and so-called atypical bacteria. These drugs are used in the treatment of respiratory, urinary, and alimentary systems infections, sexually transmitted diseases, and skin, soft tissues, bones and joints infections [1]. Besides the broad-spectrum of activity, the clinical success of fluoroquinoles is due to such features as good bioavailability after oral administration, good tissue penetration, beneficial pharmacokinetics and relatively low toxicity [2]. Even so, the global phenomenon of bacteria’s resistance to antibiotics affects fluoroquinoles as well. The first bacterial strains which demonstrated reduced sensitivity to fluoroquinolones were *Staphylococcus aureus* (including the MRSA strain) and *Pseudomonas aeruginosa* [3]. Some scientists claim that between 95% and 100% of the MRSA strains are resistant to the drugs from this group [4]. Moreover, since the 1990s, resistance to fluoroquinolones has increased also in the case of other Gram-positive and Gram-negative bacteria. For instance, the percentage of *Escherichia coli* isolates resistant to fluoroquinolones in Great Britain has increased during just five years, from 2001–2006, from 6%–20% [5]. In most cases, the rate of increased resistance is correlated with the amount of fluoroquinolones usage. Bacterial resistance may have different backgrounds, and the most frequently named mechanisms include: (i) target-site mutation; (ii) enzymatic degradation of the drug; (iii) reduced permeability of the drug; and (iv) active export of the drug through efflux pumps. However, irrespective of its molecular background, fluoroquinolone resistance compels the search for new representatives of this group characterized by strong antibacterial activity and ability to overcome bacterial resistance. As it has been proven quite recently, a novel class of fluoroquinolones obtained by molecular hybridization of ciprofloxacin and different 1,2,4-triazole derivatives demonstrate a promising antibacterial activity against both Gram-positive and Gram-negative bacteria [6]. The results showed that the chemical character of substituents connected to the 1,2,4-triazole ring affected the antibacterial activity of such compounds. The most beneficial effect was obtained when the triazole ring was connected with a hydroxyphenyl fragment. This may suggest that the hydroxyl group promotes hydrogen bonding with target enzymes. On the other hand, a disubstitution pattern of the second aryl substituent seemed to be also relevant for antibacterial potency. As the published results are quite preliminary, more detailed description of the relationship(s) between the antibacterial activity and chemical structure shall require a greater number of the synthesized derivatives. Moreover, there is no data that would explain the reasons for increased activity of 1,2,4-triazole-ciprofloxacin hybrids as compared with the activity of the ciprofloxacin alone. Therefore, to shed some light on the molecular grounds of this phenomenon, the enzymatic tests were carried out with the use of different enzymatic models obtained from *Escherichia coli* and *Staphylococcus aureus*. It was also checked if the antibacterial effect of the tested compounds resulted from selective toxicity towards bacterial cells.

## 2. Results and Discussion

### 2.1. Chemistry

**Scheme 1:**
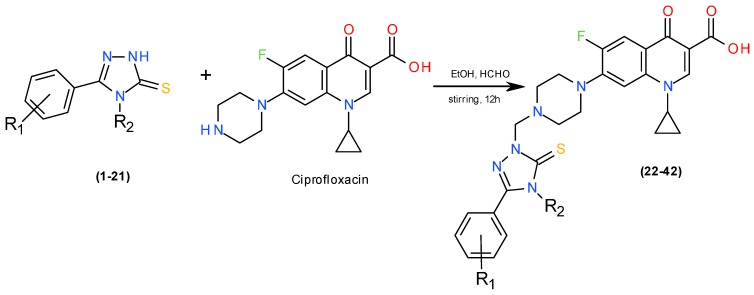
Synthetic route to compounds **22**–**42**. List of substituents: **1**, **22**. R_1_ = 2-OH, R_2_ = cyclohexyl; **2**, **23**. R_1_ = 2-OH, R_2_ = 3,4-diCl-C_6_H_3_-; **3**, **24**. R_1_ = 2-OH, R_2_ = 2,4-diCl-C_6_H_3_-; **4**, **25**. R_1_ = 2-OH, R_2_ = 3,5-diCl-C_6_H_3_-; **5**, **26**. R_1_ = 2-OH, R_2_ = 2-Cl-4-Br-C_6_H_3_-; **6**, **27**. R_1_ = 2-OH, R_2_ = 3-Cl-4-CH_3_-C_6_H_3_-; **7**, **28**. R_1_ = 2-OH, R_2_ = 3-CF_3_-4-Cl-C_6_H_3_-; **8**, **29**. R_1_ = 3-OH, R_2_ = cyclohexyl; **9**, **30**. R_1_ = 3-OH, R_2_ = 3,4-diCl-C_6_H_3_-; **10**, **31**. R_1_ = 3-OH, R_2_ = 2,4-diCl-C_6_H_3_-; **11**, **32**. R_1_ = 3-OH, R_2_ = 3,5-diCl-C_6_H_3_-; **12**, **33**. R_1_ = 3-OH, R_2_ = 2-Cl-4-Br-C_6_H_3_-; **13**, **34**. R_1_ = 3-OH, R_2_ = 3-Cl-4-CH_3_-C_6_H_3_-; **14**, **35**. R_1_ = 3-OH, R_2_ = 3-CF_3_-4-Cl-C_6_H_3_-; **15**, **36**. R_1_ = 4-OH, R_2_ = cyclohexyl; **16**, **37**. R_1_ = 4-OH, R_2_ = 3,4-diCl-C_6_H_3_-; **17**, **38**. R_1_ = 4-OH, R_2_ = 2,4-diCl-C_6_H_3_-; **18**, **39**. R_1_ = 4-OH, R_2_ = 3,5-diCl-C_6_H_3_-; **19**, **40**. R_1_ = 4-OH, R_2_ = 2-Cl-4-Br-C_6_H_3_-; **20**, **41**. R_1_ = 4-OH, R_2_ = 3-Cl-4-CH_3_-C_6_H_3_-; **21**, **42**. R_1_ = 4-OH, R_2_ = 3-CF_3_-4-Cl-C_6_H_3_-.

The title 1,2,4-triazole-ciprofloxacin hybrids were synthesized according to previously published procedure [6], in the reaction of respective 4,5-disubstituted 1,2,4-triazole-3-thiones, ciprofloxacin and formaldehyde. Substitution pattern of substituents in the 1,2,4-triazole ring is listed in Scheme 1.

### 2.2. Antibacterial Activity

The antibacterial activity of compounds (**22**–**42**) was tested on Gram-positive and Gram-negative strains. As it is shown in Table 1 and Table 2, the majority of novel hybrids obtained by a molecular hybridization of ciprofloxacin (CPX) and different 1,2,4-triazole derivatives were far more active than the initial antibiotic. What is important, the enhanced antibacterial effect concerned both Gram-positive and Gram-negative bacteria. This is even more important as the chemical modifications of fluoroquinolones resulting in the increase of activity towards one type of bacteria (e.g., Gram-positive or Gram-negative ones) usually are connected with a decrease of activity towards another type of bacteria [7,8]. It results, most probably, from the differences in the structure of the primary molecular target which, in case of Gram-negative bacteria is DNA gyrase (*gyrDNA*), and in case of Gram-positive bacteria—topoisomerase IV (*topoIV*) [9]. In the group of Gram-negative bacteria all derivatives tested demonstrated stronger than CPX effect on pathogenic strains *P. aeruginosa* and *K. pneumoniae*. The strongest inhibitory effect on the growth of *P. aeruginosa* ATCC9027 was demonstrated by the derivatives with the 2,4-disubstituted phenyl ring connected to the 1,2,4-triazole skeleton. The antibacterial activity of such derivatives was around nine times higher than CPX alone. Moreover, compound (**40**) completely inhibited the growth of *K. pneumoniae* ATCC13883 at the concentration about 18 times lower than in the case of CPX. Similar activity was also demonstrated by 3,4-dichlorophenyl derivative (**37**). Most of the synthesized compounds, moreover, demonstrated strong antibacterial effect against the remaining two Gram-negative strains (*i.e*., *E. coli* ATCC25922 and *P. mirabilis* ATCC 12453). As MIC values for Gram-negative bacteria indicate, the presence of the aromatic ring connected to the 1,2,4-triazole core (at nitrogen atom) is not required to obtain significant antibacterial activity. In most cases, respective cyclohexyl derivatives (**22**, **29**, **36**) also demonstrated stronger antibacterial effect than CPX. Similarly, the change of a hydroxyl group position (*ortho*-, *meta*-, *para*-) in another phenyl ring affected the change of activity to a slight degree only. However, its presence alone is significant, which has been confirmed by the previously obtained results [6].

**Table 1:** Antibacterial activity of compounds **22**–**42** against Gram-negative strains.

Compounds	Minimal Inhibitory Concentrations (µM)			
	*E. coli* *ATCC 25922*	*K. pneumoniae* *ATCC 13883*	*P. mirabilis* *ATCC 12453*	*P. aeruginosa* *ATCC 9027*
**22**	0.012	0.048	0.024	0.384
**23**	0.011	0.088	0.022	0.176
**24**	0.011	0.088	0.044	0.176
**25**	0.011	0.176	0.022	0.352
**26**	0.01	0.16	0.08	0.32
**27**	0.011	0.045	0.045	0.18
**28**	0.01	0.042	0.021	0.084
**29**	0.048	0.096	0.096	0.384
**30**	0.022	0.088	0.022	0.176
**31**	0.044	0.088	0.022	0.176
**32**	0.022	0.088	0.044	0.176
**33**	0.01	0.04	0.02	0.08
**34**	0.011	0.045	0.023	0.18
**35**	0.021	0.042	0.021	0.17
**36**	0.048	0.096	0.096	0.192
**37**	0.011	0.022	0.022	0.352
**38**	0.011	0.044	0.022	0.352
**39**	0.352	0.044	0.176	0.704
**40**	0.01	0.02	0.04	0.08
**41**	0.011	0.18	0.023	0.18
**42**	0.01	0.042	0.021	0.084
**CPX**	0.024	0.36	0.045	0.72

**Table 2:** Antibacterial activity of compounds **22**–**42** against Gram-positive strains

Compounds	Minimal Inhibitory Concentrations (µM)						
	MSSA-1 *	MSSA-2 **	MRSA ***	*S. epidermidis* *ATCC 12228*	*B. subtilis* *ATCC 6638*	*B. cereus* *ATCC 10876*	*M. luteus* *ATCC 10240*
**22**	1.58	0.40	0.40	0.40	0.10	0.19	3.16
**23**	0.35	0.35	0.18	0.04	0.04	0.18	1.40
**24**	0.18	0.09	0.09	0.045	0.35	0.045	1.40
**25**	0.09	0.09	0.045	0.18	0.09	0.09	0.70
**26**	0.33	0.165	0.083	0.041	0.083	0.165	1.32
**27**	0.74	0.18	0.18	0.09	0.09	0.18	1.48
**28**	0.34	0.17	0.17	0.084	0.084	0.17	1.36
**29**	3.16	1.58	1.58	0.79	0.79	0.79	6.32
**30**	0.35	0.35	0.18	0.09	0.09	0.18	1.40
**31**	0.35	0.18	0.09	0.35	0.18	0.18	1.40
**32**	0.70	0.18	0.18	0.35	0.18	0.18	1.40
**33**	0.165	0.165	0.165	0.041	0.041	0.165	1.32
**34**	0.36	0.09	0.09	0.18	0.09	0.18	0.74
**35**	0.68	0.084	0.17	0.17	0.042	0.17	1.36
**36**	3.16	1.58	1.58	1.58	0.024	0.20	3.91
**37**	0.35	0.18	0.35	0.18	0.045	0.18	0.70
**38**	0.70	0.18	0.18	0.35	0.045	0.18	1.40
**39**	5.60	1.40	2.80	0.35	0.35	0.70	22.9
**40**	0.33	0.083	0.083	0.165	0.041	0.165	0.66
**41**	0.36	0.18	0.18	0.36	0.18	0.36	0.74
**42**	0.68	0.084	0.17	0.17	0.042	0.17	1.36
**CPX**	2.96	0.72	-	1.48	0.09	0.36	5.88
**Vancomycin**	-	-	0.68	-	-	-	-

“*”–*S. aureus* ATCC 25923, “**”–*S. aureus* ATCC 6538, “***”–*S. aureus* MIKROBANK 14001.

In case of Gram-positive bacteria (Table 2), unlike the tested Gram-negative ones, the structure of the substituent attached to the nitrogen atom in the 1,2,4-triazole ring is of much greater importance. Especially in staphylococci it is clearly discernible that the presence of disubstituted phenyl ring is much more beneficial than the presence of cycloalkyl substituent. Respective cyclohexyl derivatives (**22**, **29**, **36**), although they still acted at least equally as strong as CPX, demonstrated weaker activity than other CPX-triazole hybrids. Among significant merits of the newly-obtained compounds, one should name the fact that methicillin-resistant *S. aureus* (MRSA) strain was particularly sensitive to their effect. Among *ortho*-, *meta*- and *para*-hydroxyphenyl derivatives there were compounds with at least a few times stronger antibacterial activity than vancomycin—the antibiotic used in treating heavy infections caused by MRSA. For instance, compounds **24**, **26**, **31**, **34** and **40** inhibited the growth of the tested MRSA strain about eight times stronger than vancomycin, while the antibacterial activity of 3,5-dichlorophenyl derivative (**25**) was as much as 15 times’ stronger than the activity of the previously mentioned antibiotic. The same compound (*i.e*., compound **25**) proved to be potent against other staphylococci tested, *i.e*., against MSSA-1 and *S. epidermidis*. It suggests that such a substitution pattern at the phenyl rings is beneficial from the microbiological point of view. On the other hand, the differences in activity of the respective 3,5-dichlorosubstituted derivatives (**25**, **32**, **39**) prove that the strength of antibacterial activity of CPX-triazole hybrids on staphylococci is determined by the structure of both aromatic substituents connected to the 1,2,4-triazole ring. Among the two bacilli strains used in our studies, greater clinical significance is demonstrated by the *Bacillus cereus*, since it may be the cause of dangerous food poisoning and in children it may also lead to meningitis [10,11]. In most cases the antibacterial activity of novel CPX-triazole hybrids against *B. cereus* ATCC10876 was higher than the activity of CPX alone. It is also of significance that *B. cereus* is used as a model strain in the research in relation to new drugs against anthrax as it is closely related genetically to *Bacillus anthracis*. Thus, one can assume that the compounds effective against *B. cereus* might also be equally effective in the treatment of anthrax.

### 2.3. Toxicity Evaluation

The antibacterial activity of newly synthesized compounds may be the result of their selective effect on bacterial cells or it may result from non-selective toxicity addressed to any live cells (including the human ones). In the latter case, respective compounds should be disqualified from among the potential antibacterial drugs, since the concentration which inhibits the growth of bacterial cells cannot disturb the normal functioning of host cells or tissues. In our studies, human embryonic kidney cells (HEK-293) were used to evaluate the cytotoxicity of the selected CPX-triazole hybrids, using the MTT test as a marker of cells viability Table 3). Toxicity profile of potential antibacterials may be characterized by the ratio of EC_50_/MIC [12]. The bigger the gap between EC_50_ and MIC values, the lesser the risk of toxic effects. In the cases of all investigated derivatives, (*i.e*., **23**–**28**, **33**, **34**, **39**, **40**) the median effective concentrations, *i.e.* such concentrations of the substance which inhibit cell growth in 50%, in proportion to the growth of control cells, were much higher than the concentrations causing antibacterial effect (Table 3). Due to low antibacterial activity of the tested compounds against *M. luteus* ATCC10240, the EC_50_/MIC ratio for that strain fell within the range between two and 128. On the other hand, the lowest risk of toxic effects was observed with the use of newly-obtained derivatives against *E. coli* ATCC25922 cells. In the case of this particular strain, the EC_50_ values were from 1184 to 12334 times higher than the respective MICs. The lowest toxicity against human cells HEK-293 was demonstrated by compound **28**, obtained from 4-[4-chloro-3-(trifluoromethyl)phenyl]-5-(2-hydroxyphenyl)-2,4-dihydro-3*H*-1,2,4-triazole-3-thione. When one compares its toxicity and the toxicity of compounds **23** and **27** (also possessing 3,4-disubstituted phenyl ring attached to 1,2,4-triazole core), it is clearly discernible that the effect on human cells’ viability depends rather on the presence of specific substituents in the molecule (in this case CF_3_ group) than on the substitution pattern of the phenyl ring.

**Table 3:** Toxic effects of the selected triazole-CPX hybrids towards human HEK-293 cells.

Compounds	EC_50_ ± SD (µM)	EC_50_/MIC (Range)
**23**	47.98 ± 10.61	4362–34
**24**	59.57 ± 15.92	5415–43
**25**	50.47 ± 3.95	4588–72
**26**	55.23 ± 9.74	5020–42
**27**	43.26 ± 4.28	3933–29
**28**	123.34 ± 2.67	12334–90
**33**	76.58 ± 9.74	7658–58
**34**	64.74 ± 6.41	5885–87
**39**	52.09 ± 15.08	1184–2
**40**	84.16 ± 14.61	8416–128

### 2.4. Affinity of the Selected CPX-Triazole Hybrids towards Bacterial Type II Topoisomerases

As it is already known, fluoroquinolones demonstrate affinity towards bacterial type II topoisomerases (*i.e*., DNA gyrase and topoisomerase IV). Both literature data [13], as well as the results of our research (Table 4) prove that among Gram-negative bacteria the primary molecular target for CPX is DNA gyrase. The inhibition of topoIV by CPX is only an additional mechanism conditioning the antibacterial activity of that fluoroquinolone. Whereas in case of Gram-positive bacteria, topoIV is the primary molecular target for CPX, and DNA gyrase only plays a secondary role. Taking into account the above-mentioned differences in the structure of primary molecular targets among Gram-positive and Gram-negative bacteria, the analysis of affinity of the synthesized derivatives was performed with the use of topoisomerases isolated from *S. aureus* and *E. coli*. The results presented in Table 4 show that the analyzed compounds (**24**, **25**, **39**) demonstrate other preferences as regards primary and secondary molecular targets than CPX. There is a visible weakening of affinity towards the main molecular targets with a simultaneous increase towards molecular targets deemed to be secondary. So, compounds **25** and **39** demonstrated increased affinity towards topoisomerase IV isolated from *E. coli*, whereas compounds **24** and **39** showed increased affinity towards DNA gyrase isolated from *S. aureus*. Moreover, the results of enzymatic assays have proven that stronger antibacterial activity of novel CPX derivatives (as compared to CPX alone) cannot be caused by the increased affinity towards bacterial type II topoisomerases. It seems hardly likely that such a significant decrease of affinity (e.g., from 0.15–3.5 μM for compound **24**) towards the primary molecular targets may be compensated with a slightly increased affinity to the respective secondary targets. Moreover, it looks obvious that the affinity of the synthesized compounds towards bacterial type II topoisomerases depends on the structure of both substituents connected to the 1,2,4-triazole skeleton.

**Table 4:** Affinity of the selected triazole-CPX hybrids towards bacterial type II topoisomerases obtained from *Escherichia coli* and *Staphylococcus aureus*.

	IC_50_ [μM]			
	*Escherichia coli*		*Staphylococcus aureus*	
	gyrDNA	topoIV	gyrDNA	topoIV
**24**	3.5	80.0	55.0	15.5
**25**	1.2	56.0	164.0	19.0
**39**	3.2	66.0	68.0	22.0
**Ciprofloxacin**	0.15	80.0	>100.0	4.0

The comparison of IC_50_ values for two 2-hydroxyphenyl derivatives (**24**, **25**) showed that the substitution pattern of the second phenyl ring connected to the 1,2,4-triazole ring had an important effect on the affinity to DNA gyrase, both among Gram-positive and Gram-negative bacteria. The change of location of the hydroxyl group in hydroxyphenyl moiety had also strong influence on the functioning of DNA gyrase.

Summarizing, one may state that the increased antibacterial activity of the newly synthesized CPX-triazole hybrids (**22**–**42**) is probably a sum of different factors and the changed affinity of such compounds to primary and secondary molecular targets is only one of them. Moreover, one should also consider the possibility of increased permeability of these types of compounds into bacterial cells or their reduced susceptibility to endogenous efflux systems. Possible existence of an additional, so far unknown, mechanism of antibacterial action of these compounds should not be excluded.

## 3. Experimental Section 

### 3.1. Chemistry

#### 3.1.1. General Comments

All reagents and solvents were purchased from Alfa Aesar (Ward Hill, MA, USA) and Merck Co. (Darmstadt, Germany). Melting points were determined by using Fisher-Johns apparatus (Fisher Scientific, Schwerte, Germany) and are uncorrected. The ^1^H-NMR and ^13^C-NMR spectra (in DMSO-*d*_6_) were recorded on a Bruker Avance spectrometer (Bruker BioSpin GmbH, Rheinstetten, Germany) using TMS as an internal standard. FT-IR spectra were recorded using an ATR Platinum Diamond A 225 device. Elemental analyses were performed on an AMZ 851 CHX analyzer (PG, Gdańsk, Poland) and the results were within ±0.4% of the theoretical value.

#### 3.1.2. General Procedure for the Synthesis of 1,2,4-Triazole-3-Thione Derivatives (**1**–**21**)

Compounds 1–21 were prepared by intramolecular cyclization of 1-(hydroxybenzoyl)-4-substituted thiosemicarbazides [14]. These compounds were dissolved in 2% NaOH and refluxed for 2 h. After cooling, the mixture was neutralized with 3M HCl. The precipitate formed was filtered and washed with distilled water. The compounds were crystallized from EtOH. In the cases of already known compounds, information about their properties may be retrieved in the Chemical Abstract Service database (CAS numbers are given below).

*4-Cyclohexyl-5-(2-hydroxyphenyl)-2,4-dihydro-3H-1,2,4-triazole-3-thione* (**1**). Yield: 82%. CAS: 26028-72-8.

*4-(3,4-Dichlorophenyl)-5-(2-hydroxyphenyl)-2,4-dihydro-3H-1,2,4-triazole-3-thione* (**2**). Yield: 87%, m.p. 258–260 °C, ^1^H-NMR (250 MHz): 6.77–7.74 (m, 7H, Ar-H), 10.08 (s, 1H, OH), 14.21 (s, 1H, NH). ^13^C-NMR (75 MHz): 111.30, 114.25, 117.80, 126.82, 128.68, 129.11, 129.30, 130.25, 131.18, 132.89, 148.18, 153.98, 166.00. Anal. calc. for C_14_H_9_Cl_2_N_3_OS (338.21): C 49.72, H 2.68, N 12.42. Found: C 49.58, H 2.56, N 12.27.

*4-(2,4-Dichlorophenyl)-5-(2-hydroxyphenyl)-2,4-dihydro-3H-1,2,4-triazole-3-thione* (**3**). Yield: 84%, m.p. 188–189 °C, ^1^H-NMR (250 MHz): 6.78–7.05 (m, 2H, Ar-H), 7.25–7.60 (m, 4H, Ar-H), 7.68–7.81 (m, 1H, Ar-H), 10.16 (s, 1H, OH), 14.24 (s, 1H, NH). ^13^C-NMR (75 MHz): 111.03, 114.33, 117.57, 126.52, 128.06, 129.67, 129.90, 131.18, 131.45, 131.71, 133.69, 148.39, 154.24, 166.51. Anal. calc. for C_14_H_9_Cl_2_N_3_OS (338.21): C 49.72, H 2.68, N 12.42. Found: C 49.60, H 2.53, N 12.31.

*4-(3,5-Dichlorophenyl)-5-(2-hydroxyphenyl)-2,4-dihydro-3H-1,2,4-triazole-3-thione* (**4**). Yield: 81%, m.p. 194–196 °C, ^1^H-NMR (250 MHz): 6.79–7.06 (m, 2H, Ar-H), 7.31–7.55 (m, 4H, Ar-H), 7.70–7.76 (m, 1H, Ar-H), 10.17 (s, 1H, OH), 14.24 (s, 1H, NH). ^13^C-NMR (75 MHz): 111.28, 114.33, 117.71, 125.71, 127.31, 130.31, 131.19, 132.04, 135.19, 148.09, 154.22, 166.15. Anal. calc. for C_14_H_9_Cl_2_N_3_OS (338.21): C 49.72, H 2.68, N 12.42. Found: C 49.64, H 2.50, N 12.34.

*4-(4-Bromo-2-chlorophenyl)-5-(2-hydroxyphenyl)-2,4-dihydro-3H-1,2,4-triazole-3-thione* (**5**). Yield: 80%. CAS: 896077-18-2.

*4-(3-Chloro-4-methylphenyl)-5-(2-hydroxyphenyl)-2,4-dihydro-3H-1,2,4-triazole-3-thione* (**6**). Yield: 78%, m.p. 236–238 °C, ^1^H-NMR (250 MHz): 2.34 (s, 3H, CH_3_), 6.78–6.94 (m, 2H, Ar-H), 7.12–7.18 (m, 1H, Ar-H), 7.27–7.47 (m, 4H, Ar-H), 10.08 (s, 1H, OH), 14.20 (s, 1H, NH). ^13^C-NMR (75 MHz): 17.88, 111.62, 114.20, 117.67, 125.11, 126.86, 129.59, 130.21, 130.97, 131.14, 131.83, 134.96, 148.23, 154.23, 166.15. Anal. calc. for C_15_H_12_ClN_3_OS (317.79): C 56.69, H 3.81, N 13.22. Found: C 56.45, H 3.75, N 13.02.

*4-[4-Chloro-3-(trifluoromethyl)phenyl]-5-(2-hydroxyphenyl)-2,4-dihydro-3H-1,2,4-triazole-3-thione* (**7**). Yield: 76%, m.p. 130–132 °C, ^1^H-NMR (250 MHz): 6.70–8.35 (m, 7H, Ar-H), 11.47 (s, 1H, OH), 14.20 (s, 1H, NH). ^13^C-NMR (75 MHz): 12.35, 113.72, 115.84, 117.59, 119.83, 124.71, 126.11, 128.17, 130.83, 131.95, 132.66, 134.04, 148.35, 156.82, 166.27. Anal. calc. for C_15_H_9_ClF_3_N_3_OS (371.76): C 48.46, H 2.44, N 11.30. Found: C 48.52, H 3.37, N 11.12.

*4-Cyclohexyl-5-(3-hydroxyphenyl)-2,4-dihydro-3H-1,2,4-triazole-3-thione* (**8**). Yield: 80%. CAS: 26028-79-5.

*4-(3,4-Dichlorophenyl)-5-(3-hydroxyphenyl)-2,4-dihydro-3H-1,2,4-triazole-3-thione* (**9**). Yield: 83%, m.p. 184–185 °C, ^1^H-NMR (250 MHz): 6.75–6.90 (m, 3H, Ar-H), 7.23 (t, 1H, Ar-H, *J* = 7.4 Hz), 7.44 (dd, 1H, Ar-H, *J* = 8.6 Hz, 2.4 Hz), 7.81–7.91 (m, 2H, Ar-H), 9.90 (s, 1H, OH), 14.26 (s, 1H, NH). ^13^C-NMR (75 MHz): 111.35, 114.87, 118.74, 126.92, 127.78, 129.34, 129.71, 130.71, 131.08, 132.33, 148.94, 154.49, 166.25. Anal. calc. for C_14_H_9_Cl_2_N_3_OS (338.21): C 49.72, H 2.68, N 12.42. Found: C 49.56, H 2.55, N 12.58.

*4-(2,4-Dichlorophenyl)-5-(3-hydroxyphenyl)-2,4-dihydro-3H-1,2,4-triazole-3-thione* (**10**). Yield: 80%, m.p. 196–198 °C, ^1^H-NMR (250 MHz): 6.75–6.92 (m, 3H, Ar-H), 7.23 (t, 1H, Ar-H, *J* = 7.9 Hz), 7.68–7.82 (m, 2H, Ar-H), 7.96 (d, 1H, Ar-H, *J* = 2.2 Hz), 9.94 (s, 1H, OH), 14.22 (s, 1H, NH). ^13^C-NMR (75 MHz): 111.20, 114.38, 117.90, 126.71, 127.93, 129.27, 130.72, 131.37, 131.83, 132.12, 133.59, 148.63, 154.82, 166.05. Anal. calc. for C_14_H_9_Cl_2_N_3_OS (338.21): C 49.72, H 2.68, N 12.42. Found: C 49.61, H 2.49, N 12.30.

*4-(3,5-Dichlorophenyl)-5-(3-hydroxyphenyl)-2,4-dihydro-3H-1,2,4-triazole-3-thione* (**11**). Yield: 85%, m.p. 296–298 °C, ^1^H-NMR (250 MHz): 6.73–6.93 (m, 3H, Ar-H), 7.23 (t, 1H, Ar-H, *J* = 7.8 Hz), 7.62–7.68 (m, 2H, Ar-H), 7.79–7.86 (m, 1H, Ar-H), 9.87 (s, 1H, OH), 14.24 (s, 1H, NH). ^13^C-NMR (75 MHz): 113.72, 116.19, 117.70, 125.07, 126.78, 128.06, 128.50, 132.85, 135.39, 148.86, 155.75, 166.91. Anal. calc. for C_14_H_9_Cl_2_N_3_OS (338.21): C 49.72, H 2.68, N 12.42. Found: C 49.85, H 2.50, N 12.29.

*4-(4-Bromo-2-chlorophenyl)-5-(3-hydroxyphenyl)-2,4-dihydro-3H-1,2,4-triazole-3-thione* (**12**). Yield: 77%, m.p. 204–206 °C, ^1^H-NMR (250 MHz): 6.74–6.93 (m, 3H, Ar-H), 7.23 (t, 1H, Ar-H, *J* = 7.7 Hz), 7.65–7.88 (m, 2H, Ar-H), 8.09 (d, 1H, Ar-H, *J* = 2.6 Hz), 9.95 (s, 1H, OH), 14.21 (s, 1H, NH). Anal. calc. for C_14_H_9_BrClN_3_OS (382.66): C 43.94, H 2.37, N 10.98. Found: C 43.81, H 2.50, N 11.13.

*4-(3-Chloro-4-methylphenyl)-5-(3-hydroxyphenyl)-2,4-dihydro-3H-1,2,4-triazole-3-thione* (**13**). Yield: 85%, m.p. 212–214 °C, ^1^H-NMR (250 MHz): 2.43 (s, 3H, CH_3_), 6.72–6.90 (m, 3H, Ar-H), 7.14–7.28 (m, 2H, Ar-H), 7.50 (d, 1H, Ar-H, *J* = 8.0 Hz), 7.60 (d, 1H, Ar-H, *J* = 2.2 Hz), 9.87 (s, 1H, OH), 14.15 (s, 1H, NH). ^13^C-NMR (75 MHz): 18.03, 113.73, 116.11, 117.60, 125.32, 126.09, 127.64, 128.37, 130.31, 131.94, 132.05, 135.65, 149.07, 155.73, 167.04. Anal. calc. for C_15_H_12_ClN_3_OS (317.79): C 56.69, H 3.81, N 13.22. Found: C 56.82, H 3.86, N 13.02.

*4-[4-Chloro-3-(trifluoromethyl)phenyl]-5-(3-hydroxyphenyl)-2,4-dihydro-3H-1,2,4-triazole-3-thione* (**14**). Yield: 80%, m.p. 154–156 °C, ^1^H-NMR (250 MHz): 6.72–6.91 (m, 3H, Ar-H), 7.22 (t, 1H, Ar-H, *J* = 7.8 Hz), 7.70–8.10 (m, 3H, Ar-H), 9.89 (s, 1H, OH), 14.26 (s, 1H, NH). ^13^C-NMR (75 MHz): 12.46, 113.89, 116.14, 117.82, 119.15, 124.17, 125.10, 128.45, 130.65, 131.28, 132.50, 133.14, 148.98, 155.74, 166.20. Anal. calc. for C_15_H_9_ClF_3_N_3_OS (371.76): C 48.46, H 2.44, N 11.30. Found: C 48.59, H 2.31, N 11.42.

*4-Cyclohexyl-5-(4-hydroxyphenyl)-2,4-dihydro-3H-1,2,4-triazole-3-thione* (**15**). Yield: 76%. CAS: 26028-87-5.

*4-(3,4-Dichlorophenyl)-5-(4-hydroxyphenyl)-2,4-dihydro-3H-1,2,4-triazole-3-thione* (**16**). Yield: 87%, m.p. 232–234 °C, ^1^H-NMR (250 MHz): 6.80 (dd, 2H, Ar-H, *J* = 6.7 Hz, 2.0 Hz), 7.20 (dd, 2H, Ar-H, *J* = 6.7 Hz, 2.1 Hz), 7.41 (dd, 1H, Ar-H, *J* = 8.6 Hz, 2.4 Hz), 7.62–7.89 (m, 2H, Ar-H), 11.37 (s, 1H, OH), 14.15 (s, 1H, NH). ^13^C-NMR (75 MHz): 111.48, 114.27, 119.22, 126.32, 127.78, 129.32, 129.93, 131.11, 131.84, 132.63, 148.74, 154.56, 166.52. Anal. calc. for C_14_H_9_Cl_2_N_3_OS (338.21): C 49.72, H 2.68, N 12.42. Found: C 49.67, H 2.45, N 12.33.

*4-(2,4-Dichlorophenyl)-5-(4-hydroxyphenyl)-2,4-dihydro-3H-1,2,4-triazole-3-thione* (**17**). Yield: 83%, m.p. 257–258 °C, ^1^H-NMR (250 MHz): 6.72 (dd, 2H, Ar-H, *J* = 6.7 Hz, 2.1 Hz), 7.14 (dd, 2H, Ar-H, *J* = 6.7 Hz, 2.1 Hz), 7.60–7.72 (m, 2H, Ar-H), 7.86 (d, 1H, Ar-H, *J* = 2.1 Hz), 10.11 (s, 1H, OH), 14.11 (s, 1H, NH). ^13^C-NMR (75 MHz): 114.22, 114.60, 127.41, 127.88, 128.60, 130.13, 131.82, 134.18, 149.16, 158.06, 166.76. Anal. calc. for C_14_H_9_Cl_2_N_3_OS (338.21): C 49.72, H 2.68, N 12.42. Found: C 49.57, H 2.64, N 12.59.

*4-(3,5-Dichlorophenyl)-5-(4-hydroxyphenyl)-2,4-dihydro-3H-1,2,4-triazole-3-thione* (**18**). Yield: 82%, m.p. 258–260 °C, ^1^H-NMR (250 MHz): 6.81 (dd, 2H, Ar-H, *J* = 6.7 Hz, 2.1 Hz), 7.22 (dd, 2H, Ar-H, *J* = 6.7 Hz, 2.1 Hz), 7.63 (d, 2H, Ar-H, *J* = 1.9 Hz), 7.84 (t, 1H, Ar-H, *J* = 1.9 Hz), 10.17 (s, 1H, OH), 14.15 (s, 1H, NH). ^13^C-NMR (75 MHz): 114.04, 114.55, 126.84, 127.95, 128.72, 132.82, 135.51, 149.14, 157.83, 166.57. Anal. calc. for C_14_H_9_Cl_2_N_3_OS (338.21): C 49.72, H 2.68, N 12.42. Found: C 49.89, H 2.58, N 12.59.

*4-(4-Bromo-2-chlorophenyl)-5-(4-hydroxyphenyl)-2,4-dihydro-3H-1,2,4-triazole-3-thione* (**19**). Yield: 76%, m.p. 262–263 °C, ^1^H-NMR (250 MHz): 6.79 (dd, 2H, Ar-H, *J* = 6.7 Hz, 1.9 Hz), 7.22 (dd, 2H, Ar-H, *J* = 6.7 Hz, 2.0 Hz), 7.69 (d, 1H, Ar-H, *J* = 8.4 Hz), 7.80–7.87 (m, 1H, Ar-H), 8.05 (d, 1H, Ar-H, *J* = 2.1 Hz), 10.20 (s, 1H, OH), 14.18 (s, 1H, NH). Anal. calc. for C_14_H_9_BrClN_3_OS (382.66): C 43.94, H 2.37, N 10.98. Found: C 44.13, H 2.28, N 10.78.

*4-(3-Chloro-4-methylphenyl)-5-(4-hydroxyphenyl)-2,4-dihydro-3H-1,2,4-triazole-3-thione* (**20**). Yield: 78%, m.p. 263–264 °C, ^1^H-NMR (250 MHz): 2.47 (s, 3H, CH_3_), 6.77 (dd, 2H, Ar-H, *J* = 6.7 Hz, 2.1 Hz), 7.20 (dd, 2H, Ar-H, *J* = 6.7 Hz, 2.1 Hz), 7.26 (d, 1H, Ar-H, *J* = 2.1 Hz), 7.48–7.60 (m, 2H, Ar-H), 10.14 (s, 1H, OH), 14.09 (s, 1H, NH). ^13^C-NMR (75 MHz): 18.01, 113.98, 114.84, 126.22, 127.70, 128.60, 130.27, 131.90, 132.18, 135.62, 149.33, 157.76, 166.74. Anal. calc. for C_15_H_12_ClN_3_OS (317.79): C 56.69, H 3.81, N 13.22. Found: C 56.51, H 3.60, N 13.04.

*4-[4-Chloro-3-(trifluoromethyl)phenyl]-5-(4-hydroxyphenyl)-2,4-dihydro-3H-1,2,4-triazole-3-thione* (**21**). Yield: 74%, m.p. 160–162 °C, ^1^H-NMR (250 MHz): 6.83–6.97 (m, 3H, Ar-H), 7.20 (t, 1H, Ar-H, *J* = 7.9 Hz), 7.65–7.92 (m, 3H, Ar-H), 10.17 (s, 1H, OH), 14.05 (s, 1H, NH). Anal. calc. for C_15_H_9_ClF_3_N_3_OS (371.76): C 48.46, H 2.44, N 11.30. Found: C 48.59, H 2.30, N 11.43.

#### 3.1.3. General Procedure for the Synthesis of 1,2,4-Triazole-Ciprofloxacin Hybrids (**22**–**42**)

1 mmol of the respective 1,2,4-triazole derivative (**1**–**21**) was dissolved (with heating) in 40 mL of anhydrous ethanol and then the equimolar amount of ciprofloxacin and formaldehyde solution were added. The obtained suspension was stirred at room temperature for 12 hours. The precipitate formed was filtered off, dried, and crystallized from ethanol to give compounds **22**–**42**.

*1-Cyclopropyl-6-fluoro-7-[4-{[4-cyclohexyl-3-(2-hydroxyphenyl)-5-thioxo-4,5-dihydro-1H-1,2,4-triazol-1-yl]methyl}piperazin-1-yl]-4-oxo-1,4-dihydroquinoline-3-carboxylic acid* (**22**). Yield: 70%, m.p. 128–130 °C, ^1^H-NMR (250 MHz): 1.06–2.56 (m, 15H, cyclopropyl + cyclohexyl), 2.89 (bs, 4H, piperazine), 3.32 (bs, 4H, piperazine), 3.81–3.85 (m, 1H, cyclopropyl), 4.46 (s, 2H, CH_2_), 7.06–7.65 (m, 6H, Ar-H), 8.61 (s, 1H, Ar-H), 10.16 (s, 1H, OH), 14.92 (s, 1H, COOH). IR (ATR): 3454, 3326 (O-H), 3062, 2962, 2861 (C-H), 1727 (C=O), 1620 (C=N), 1419 (C-O), 1324 (C=S), 1264 (O-H), 1043 (C-F). Anal. calc. for C_32_H_35_FN_6_O_4_S (618.72): C 62.12, H 5.70, N 13.58. Found: C 62.03, H 5.91, N 13.40.

*1-Cyclopropyl-6-fluoro-7-[4-{[4-(3,4-dichlorophenyl)-3-(2-hydroxyphenyl)-5-thioxo-4,5-dihydro-1H-1,2,4-triazol-1-yl]methyl}piperazin-1-yl]-4-oxo-1,4-dihydroquinoline-3-carboxylic acid* (**23**). Yield: 68%, m.p. 237–238 °C, ^1^H-NMR (250 MHz): 1.12–1.14 (m, 2H, cyclopropyl), 1.20–1.24 (m, 2H, cyclopropyl), 2.85 (bs, 4H, piperazine), 3.34 (bs, 4H, piperazine), 3.82–3.86 (m, 1H, cyclopropyl), 4.47 (s, 2H, CH_2_), 6.84–7.68 (m, 9H, Ar-H), 8.65 (s, 1H, Ar-H), 10.12 (s, 1H, OH), 15.02 (s, 1H, COOH). IR (ATR): 3482 (O-H), 3122, 2905 (C-H), 1747 (C=O), 1613 (C=N), 1423 (C-O), 1310 (C=S), 1253 (O-H), 1011 (C-F). Anal. calc. for C_32_H_27_Cl_2_FN_6_O_4_S (681.56): C 56.39, H 3.99, N 12.33. Found: C 56.56, H 4.18, N 12.05.

*1-Cyclopropyl-6-fluoro-7-[4-{[4-(2,4-dichlorophenyl)-3-(2-hydroxyphenyl)-5-thioxo-4,5-dihydro-1H-1,2,4-triazol-1-yl]methyl}piperazin-1-yl]-4-oxo-1,4-dihydroquinoline-3-carboxylic acid* (**24**). Yield: 72%, m.p. 220–221 °C, ^1^H-NMR (250 MHz): 1.08–1.12 (m, 2H, cyclopropyl), 1.18–1.22 (m, 2H, cyclopropyl), 2.86 (bs, 4H, piperazine), 3.32 (bs, 4H, piperazine), 3.81–3.84 (m, 1H, cyclopropyl), 4.52 (s, 2H, CH_2_), 6.87-7.68 (m, 9H, Ar-H), 8.68 (s, 1H, Ar-H), 10.21 (s, 1H, OH), 14.95 (s, 1H, COOH). IR (ATR): 3480 (O-H), 3057, 2933 (C-H), 1722 (C=O), 1633 (C=N), 1429 (C-O), 1328 (C=S), 1258 (O-H), 1018 (C-F). Anal. calc. for C_32_H_27_Cl_2_FN_6_O_4_S (681.56): C 56.39, H 3.99, N 12.33. Found: C 56.59, H 3.86, N 12.25.

*1-Cyclopropyl-6-fluoro-7-[4-{[4-(3,5-dichlorophenyl)-3-(2-hydroxyphenyl)-5-thioxo-4,5-dihydro-1H-1,2,4-triazol-1-yl]methyl}piperazin-1-yl]-4-oxo-1,4-dihydroquinoline-3-carboxylic acid* (**25**). Yield: 73%, m.p. 224–226 °C, ^1^H-NMR (250 MHz): 1.15–1.18 (m, 2H, cyclopropyl), 1.25–1.29 (m, 2H, cyclopropyl), 2.80 (bs, 4H, piperazine), 3.35 (bs, 4H, piperazine), 3.80–3.84 (m, 1H, cyclopropyl), 4.44 (s, 2H, CH_2_), 6.94–7.75 (m, 9H, Ar-H), 8.68 (s, 1H, Ar-H), 10.18 (s, 1H, OH), 15.02 (s, 1H, COOH). IR (ATR): 3437 (O-H), 2983, 2835 (C-H), 1725 (C=O), 1643 (C=N), 1432 (C-O), 1310 (C=S), 1254 (O-H), 1041 (C-F). Anal. calc. for C_32_H_27_Cl_2_FN_6_O_4_S (681.56): C 56.39, H 3.99, N 12.33. Found: C 56.50, H 4.10, N 12.42.

*1-Cyclopropyl-6-fluoro-7-[4-{[4-(4-bromo-2-chlorophenyl)-3-(2-hydroxyphenyl)-5-thioxo-4,5-dihydro-1H-1,2,4-triazol-1-yl]methyl}piperazin-1-yl]-4-oxo-1,4-dihydroquinoline-3-carboxylic acid* (**26**). Yield: 66%, m.p. 214–216 °C, ^1^H-NMR (250 MHz): 1.20–1.23 (m, 2H, cyclopropyl), 1.28–1.32 (m, 2H, cyclopropyl), 2.80 (s, 4H, piperazine), 3.41 (s, 4H, piperazine), 3.81–3.85 (m, 1H, cyclopropyl), 4.56 (s, 2H, CH_2_), 6.86–7.74 (m, 9H, Ar-H), 8.68 (s, 1H, Ar-H), 10.21 (s, 1H, OH), 14.85 (s, 1H, COOH). IR (ATR): 3473, 3364 (O-H), 3084, 2872 (C-H), 1724 (C=O), 1626 (C=N), 1432 (C-O), 1325 (C=S), 1272 (O-H), 1008 (C-F). Anal. calc. for C_32_H_27_BrClFN_6_O_4_S (726.01): C 52.94, H 3.75, N 11.58. Found: C 52.82, H 3.61, N 11.70.

*1-Cyclopropyl-6-fluoro-7-[4-{[4-(3-chloro-4-methylphenyl)-3-(2-hydroxyphenyl)-5-thioxo-4,5-dihydro-1H-1,2,4-triazol-1-yl]methyl}piperazin-1-yl]-4-oxo-1,4-dihydroquinoline-3-carboxylic acid* (**27**). Yield: 75%, m.p. 237–238 °C, ^1^H-NMR (250 MHz): 1.12–1.14 (m, 2H, cyclopropyl), 1.21–1.23 (m, 2H, cyclopropyl), 2.24 (s, 3H, CH_3_), 2.92 (bs, 4H, piperazine), 3.41 (bs, 4H, piperazine), 3.82-3.85 (m, 1H, cyclopropyl), 4.51 (s, 2H, CH_2_), 6.87-7.68 (m, 9H, Ar-H), 8.60 (s, 1H, Ar-H), 10.19 (s, 1H, OH), 15.00 (s, 1H, COOH). IR (ATR): 3469 (O-H), 3085, 2958, 2852 (C-H), 1740 (C=O), 1634 (C=N), 1419 (C-O), 1320 (C=S), 1276 (O-H), 1033 (C-F). Anal. calc. for C_33_H_30_ClFN_6_O_4_S (661.14): C 59.95, H 4.57, N 12.71. Found: C 60.12, H 4.53, N 12.79.

*1-Cyclopropyl-6-fluoro-7-[4-{[4-(4-chloro-3-trifluoromethylphenyl)-3-(2-hydroxyphenyl)-5-thioxo-4,5-dihydro-1H-1,2,4-triazol-1-yl]methyl}piperazin-1-yl]-4-oxo-1,4-dihydroquinoline-3-carboxylic acid* (**28**). Yield: 63%, m.p. 230–231 °C, ^1^H-NMR (250 MHz): 1.17–1.20 (m, 2H, cyclopropyl), 1.27–1.31 (m, 2H, cyclopropyl), 2.90 (s, 4H, piperazine), 3.31 (s, 4H, piperazine), 3.82–3.86 (m, 1H, cyclopropyl), 4.58 (s, 2H, CH_2_), 6.79–7.72 (m, 9H, Ar-H), 8.66 (s, 1H, Ar-H), 10.18 (s, 1H, OH), 14.89 (s, 1H, COOH). IR (ATR): 3459 (O-H), 3103, 3015, 2891 (C-H), 1729 (C=O), 1631 (C=N), 1429 (C-O), 1323 (C=S), 1274 (O-H), 1046 (C-F). Anal. calc. for C_33_H_27_ClF_4_N_6_O_4_S (715.12): C 55.42, H 3.81, N 11.75. Found: C 55.63, H 3.70, N 11.91.

*1-Cyclopropyl-6-fluoro-7-[4-{[4-cyclohexyl-3-(3-hydroxyphenyl)-5-thioxo-4,5-dihydro-1H-1,2,4-triazol-1-yl]methyl}piperazin-1-yl]-4-oxo-1,4-dihydroquinoline-3-carboxylic acid* (**29**). Yield: 71%, m.p. 246–248 °C, ^1^H-NMR (250 MHz): 1.11–2.68 (m, 15H, cyclopropyl + cyclohexyl), 2.84 (s, 4H, piperazine), 3.36 (bs, 4H, piperazine), 3.82–3.87 (m, 1H, cyclopropyl), 4.54 (s, 2H, CH_2_), 6.94–7.58 (m, 6H, Ar-H), 8.66 (s, 1H, Ar-H), 9.87 (s, 1H, OH), 14.97 (s, 1H, COOH). IR (ATR): 3506, 3365 (O-H), 3050, 2962, 2901 (C-H), 1743 (C=O), 1621 (C=N), 1416 (C-O), 1315 (C=S), 1279 (O-H), 1026 (C-F). Anal. calc. for C_32_H_35_FN_6_O_4_S (618.72): C 62.12, H 5.70, N 13.58. Found: C 62.19, H 5.84, N 13.71.

1*-Cyclopropyl-6-fluoro-7-[4-{[4-(3,4-dichlorophenyl)-3-(3-hydroxyphenyl)-5-thioxo-4,5-dihydro-1H-1,2,4-triazol-1-yl]methyl}piperazin-1-yl]-4-oxo-1,4-dihydroquinoline-3-carboxylic acid* (**30**). Yield: 70%, m.p. 214–216 °C, ^1^H-NMR (250 MHz): 1.13–1.17 (m, 2H, cyclopropyl), 1.24–1.28 (m, 2H, cyclopropyl), 2.84 (bs, 4H, piperazine), 3.36 (bs, 4H, piperazine), 3.82–3.87 (m, 1H, cyclopropyl), 4.49 (s, 2H, CH_2_), 6.84–7.75 (m, 9H, Ar-H), 8.65 (s, 1H, Ar-H), 9.87 (s, 1H, OH), 14.89 (s, 1H, COOH). IR (ATR): 3454 (O-H), 3105, 3023, 2882 (C-H), 1726 (C=O), 1631 (C=N), 1433 (C-O), 1307 (C=S), 1271 (O-H), 1039 (C-F). Anal. calc. for C_32_H_27_Cl_2_FN_6_O_4_S (681.56): C 56.39, H 3.99, N 12.33. Found: C 56.57, H 4.14, N 12.27.

*1-Cyclopropyl-6-fluoro-7-[4-{[4-(2,4-dichlorophenyl)-3-(3-hydroxyphenyl)-5-thioxo-4,5-dihydro-1H-1,2,4-triazol-1-yl]methyl}piperazin-1-yl]-4-oxo-1,4-dihydroquinoline-3-carboxylic acid* (**31**). Yield: 78%, spectral data published in Ref. [6]

*1-Cyclopropyl-6-fluoro-7-[4-{[4-(3,5-dichlorophenyl)-3-(3-hydroxyphenyl)-5-thioxo-4,5-dihydro-1H-1,2,4-triazol-1-yl]methyl}piperazin-1-yl]-4-oxo-1,4-dihydroquinoline-3-carboxylic acid* (**32**). Yield: 74%, spectral data published in Ref. [6]

*1-Cyclopropyl-6-fluoro-7-[4-{[4-(4-bromo-2-chlorophenyl)-3-(3-hydroxyphenyl)-5-thioxo-4,5-dihydro-1H-1,2,4-triazol-1-yl]methyl}piperazin-1-yl]-4-oxo-1,4-dihydroquinoline-3-carboxylic acid* (**33**). Yield: 67%, m.p. 246–248 °C, ^1^H-NMR (250 MHz): 1.12–1.17 (m, 2H, cyclopropyl), 1.21–1.24 (m, 2H, cyclopropyl), 2.89 (bs, 4H, piperazine), 3.32 (bs, 4H, piperazine), 3.82–3.86 (m, 1H, cyclopropyl), 4.47 (s, 2H, CH_2_), 6.95–7.84 (m, 9H, Ar-H), 8.62 (s, 1H, Ar-H), 9.93 (s, 1H, OH), 14.82 (s, 1H, COOH). IR (ATR): 3469 (O-H), 3005, 2950 (C-H), 1720 (C=O), 1631 (C=N), 1427 (C-O), 1328 (C=S), 1256 (O-H), 1027 (C-F). Anal. calc. for C_32_H_27_BrClFN_6_O_4_S (726.01): C 52.94, H 3.75, N 11.58. Found: C 53.12, H 3.72, N 11.42.

*1-Cyclopropyl-6-fluoro-7-[4-{[4-(3-chloro-4-methylphenyl)-3-(3-hydroxyphenyl)-5-thioxo-4,5-dihydro-1H-1,2,4-triazol-1-yl]methyl}piperazin-1-yl]-4-oxo-1,4-dihydroquinoline-3-carboxylic acid* (**34**). Yield: 73%, m.p. 204–206 °C, ^1^H-NMR (250 MHz): 1.16–1.20 (m, 2H, cyclopropyl), 1.25–1.28 (m, 2H, cyclopropyl), 2.41 (s, 3H, CH_3_), 2.72 (bs, 4H, piperazine), 3.32 (bs, 4H, piperazine), 3.80–3.85 (m, 1H, cyclopropyl), 4.46 (s, 2H, CH_2_), 6.86–7.75 (m, 9H, Ar-H), 8.70 (s, 1H, Ar-H), 9.84 (s, 1H, OH), 14.96 (s, 1H, COOH). IR (ATR): 3504, 3375 (O-H), 3042, 2910 (C-H), 1711 (C=O), 1627 (C=N), 1441 (C-O), 1317 (C=S), 1250 (O-H), 1022 (C-F). Anal. calc. for C_33_H_30_ClFN_6_O_4_S (661.14): C 59.95, H 4.57, N 12.71. Found: C 59.78, H 4.62, N 12.82.

*1-Cyclopropyl-6-fluoro-7-[4-{[4-(4-chloro-3-trifluoromethylphenyl)-3-(3-hydroxyphenyl)-5-thioxo-4,5-dihydro-1H-1,2,4-triazol-1-yl]methyl}piperazin-1-yl]-4-oxo-1,4-dihydroquinoline-3-carboxylic acid* (**35**). Yield: 63%, m.p. 168–170 °C, ^1^H-NMR (250 MHz): 1.20–1.24 (m, 2H, cyclopropyl), 1.28–1.31 (m, 2H, cyclopropyl), 2.90 (bs, 4H, piperazine), 3.35 (bs, 4H, piperazine), 3.83-3.86 (m, 1H, cyclopropyl), 4.67 (s, 2H, CH_2_), 6.87–7.63 (m, 9H, Ar-H), 8.69 (s, 1H, Ar-H), 9.81 (s, 1H, OH), 14.73 (s, 1H, COOH). IR (ATR): 3483 (O-H), 2982, 2927, 2831 (C-H), 1730 (C=O), 1642 (C=N), 1437 (C-O), 1319 (C=S), 1272 (O-H), 1023 (C-F). Anal. calc. for C_33_H_27_ClF_4_N_6_O_4_S (715.12): C 55.42, H 3.81, N 11.75. Found: C 55.49, H 3.69, N 11.64.

*1-Cyclopropyl-6-fluoro-7-[4-{[4-cyclohexyl-3-(4-hydroxyphenyl)-5-thioxo-4,5-dihydro-1H-1,2,4-triazol-1-yl]methyl}piperazin-1-yl]-4-oxo-1,4-dihydroquinoline-3-carboxylic acid* (**36**). Yield: 74%, m.p. 256–258 °C, ^1^H-NMR (250 MHz): 1.01–2.60 (m, 15H, cyclopropyl + cyclohexyl), 2.92 (bs, 4H, piperazine), 3.30 (bs, 4H, piperazine), 3.81–3.87 (m, 1H, cyclopropyl), 4.62 (s, 2H, CH_2_), 6.98–7.53 (m, 6H, Ar-H), 8.63 (s, 1H, Ar-H), 9.95 (s, 1H, OH), 15.06 (s, 1H, COOH). IR (ATR): 3512 (O-H), 3100, 2944, 2875 (C-H), 1730 (C=O), 1621 (C=N), 1417 (C-O), 1316 (C=S), 1254 (O-H), 1046 (C-F). Anal. calc. for C_32_H_35_FN_6_O_4_S (618.72): C 62.12, H 5.70, N 13.58. Found: C 62.21, H 5.54, N 13.42.

*1-Cyclopropyl-6-fluoro-7-[4-{[4-(3,4-dichlorophenyl)-3-(4-hydroxyphenyl)-5-thioxo-4,5-dihydro-1H-1,2,4-triazol-1-yl]methyl}piperazin-1-yl]-4-oxo-1,4-dihydroquinoline-3-carboxylic acid* (**37**). Yield: 73%, m.p. 205–206 °C, ^1^H-NMR (250 MHz): 1.16–1.20 (m, 2H, cyclopropyl), 1.27–1.31 (m, 2H, cyclopropyl), 2.74 (bs, 4H, piperazine), 3.28 (bs, 4H, piperazine), 3.82–3.86 (m, 1H, cyclopropyl), 4.68 (s, 2H, CH_2_), 6.83–7.76 (m, 9H, Ar-H), 8.73 (s, 1H, Ar-H), 10.74 (s, 1H, OH), 14.92 (s, 1H, COOH). IR (ATR): 3495 (O-H), 2972, 2869 (C-H), 1741 (C=O), 1628 (C=N), 1423 (C-O), 1329 (C=S), 1260 (O-H), 1038 (C-F). Anal. calc. for C_32_H_27_Cl_2_FN_6_O_4_S (681.56): C 56.39, H 3.99, N 12.33. Found: C 56.51, H 4.10, N 12.23.

*1-Cyclopropyl-6-fluoro-7-[4-{[4-(2,4-dichlorophenyl)-3-(4-hydroxyphenyl)-5-thioxo-4,5-dihydro-1H-1,2,4-triazol-1-yl]methyl}piperazin-1-yl]-4-oxo-1,4-dihydroquinoline-3-carboxylic acid* (**38**). Yield: 68%, m.p. 234–236 °C, ^1^H-NMR (250 MHz): 1.20–1.124 (m, 2H, cyclopropyl), 1.33–1.36 (m, 2H, cyclopropyl), 2.94 (s, 4H, piperazine), 3.42 (s, 4H, piperazine), 3.81–3.85 (m, 1H, cyclopropyl), 4.56 (s, 2H, CH_2_), 6.84–7.76 (m, 9H, Ar-H), 8.69 (s, 1H, Ar-H), 10.18 (s, 1H, OH), 15.03 (s, 1H, COOH). IR (ATR): 3479 (O-H), 3025, 2902, 2872 (C-H), 1738 (C=O), 1627 (C=N), 1419 (C-O), 1324 (C=S), 1259 (O-H), 1027 (C-F). Anal. calc. for C_32_H_27_Cl_2_FN_6_O_4_S (681.56): C 56.39, H 3.99, N 12.33. Found: C 56.50, H 3.83, N 12.37.

*1-Cyclopropyl-6-fluoro-7-[4-{[4-(3,5-dichlorophenyl)-3-(4-hydroxyphenyl)-5-thioxo-4,5-dihydro-1H-1,2,4-triazol-1-yl]methyl}piperazin-1-yl]-4-oxo-1,4-dihydroquinoline-3-carboxylic acid* (**39**). Yield: 64%, m.p. 254–256 °C, ^1^H-NMR (250 MHz): 1.15–1.19 (m, 2H, cyclopropyl), 1.28–1.31 (m, 2H, cyclopropyl), 2.99 (bs, 4H, piperazine), 3.38 (bs, 4H, piperazine), 3.83–3.86 (m, 1H, cyclopropyl), 4.68 (s, 2H, CH_2_), 6.91–7.77 (m, 9H, Ar-H), 8.60 (s, 1H, Ar-H), 10.21 (s, 1H, OH), 14.76 (s, 1H, COOH). IR (ATR): 3518 (O-H), 3075, 2942 (C-H), 1717 (C=O), 1629 (C=N), 1456 (C-O), 1314 (C=S), 1272 (O-H), 1016 (C-F). Anal. calc. for C_32_H_27_Cl_2_FN_6_O_4_S (681.56): C 56.39, H 3.99, N 12.33. Found: C 56.47, H 3.80, N 12.43.

*1-Cyclopropyl-6-fluoro-7-[4-{[4-(4-bromo-2-chlorophenyl)-3-(4-hydroxyphenyl)-5-thioxo-4,5-dihydro-1H-1,2,4-triazol-1-yl]methyl}piperazin-1-yl]-4-oxo-1,4-dihydroquinoline-3-carboxylic acid* (**40**). Yield: 67%, m.p. 242–244 °C, ^1^H-NMR (250 MHz): 1.23–1.27 (m, 2H, cyclopropyl), 1.35–1.38 (m, 2H, cyclopropyl), 2.84 (bs, 4H, piperazine), 3.39 (bs, 4H, piperazine), 3.78–3.83 (m, 1H, cyclopropyl), 4.60 (s, 2H, CH_2_), 6.85–7.69 (m, 9H, Ar-H), 8.73 (s, 1H, Ar-H), 10.20 (s, 1H, OH), 14.87 (s, 1H, COOH). IR (ATR): 3450 (O-H), 3122, 2952, 2920 (C-H), 1720 (C=O), 1634 (C=N), 1438 (C-O), 1320 (C=S), 1273 (O-H), 1033 (C-F). Anal. calc. for C_32_H_27_BrClFN_6_O_4_S (726.01): C 52.94, H 3.75, N 11.58. Found: C 53.08, H 3.72, N 11.42.

*1-Cyclopropyl-6-fluoro-7-[4-{[4-(3-chloro-4-methylphenyl)-3-(4-hydroxyphenyl)-5-thioxo-4,5-dihydro-1H-1,2,4-triazol-1-yl]methyl}piperazin-1-yl]-4-oxo-1,4-dihydroquinoline-3-carboxylic acid* (**41**). Yield: 72%, m.p. 217–219 °C, ^1^H-NMR (250 MHz): 1.14–1.18 (m, 2H, cyclopropyl), 1.30–1.35 (m, 2H, cyclopropyl), 2.47 (s, 3H, CH_3_), 2.96 (s, 4H, piperazine), 3.43 (s, 4H, piperazine), 3.82–3.86 (m, 1H, cyclopropyl), 4.66 (s, 2H, CH_2_), 6.93–7.76 (m, 9H, Ar-H), 8.68 (s, 1H, Ar-H), 10.11 (s, 1H, OH), 14.96 (s, 1H, COOH). IR (ATR): 3506 (O-H), 3005, 2948, 2781 (C-H), 1740 (C=O), 1638 (C=N), 1441 (C-O), 1341 (C=S), 1264 (O-H), 1018 (C-F). Anal. calc. for C_33_H_30_ClFN_6_O_4_S (661.14): C 59.95, H 4.57, N 12.71. Found: C 59.84, H 4.72, N 12.65.

*1-Cyclopropyl-6-fluoro-7-[4-{[4-(4-chloro-3-trifluoromethylphenyl)-3-(4-hydroxyphenyl)-5-thioxo-4,5-dihydro-1H-1,2,4-triazol-1-yl]methyl}piperazin-1-yl]-4-oxo-1,4-dihydroquinoline-3-carboxylic acid* (**42**). Yield: 63%, m.p. 198–200 °C, ^1^H-NMR (250 MHz): 1.20–1.124 (m, 2H, cyclopropyl), 1.34–1.38 (m, 2H, cyclopropyl), 2.87 (s, 4H, piperazine), 3.38 (s, 4H, piperazine), 3.82–3.86 (m, 1H, cyclopropyl), 4.57 (s, 2H, CH_2_), 6.90–7.76 (m, 9H, Ar-H), 8.66 (s, 1H, Ar-H), 10.04 (s, 1H, OH), 14.94 (s, 1H, COOH). IR (ATR): 3487 (O-H), 2984, 2847 (C-H), 1724 (C=O), 1619 (C=N), 1438 (C-O), 1317 (C=S), 1269 (O-H), 1030 (C-F). Anal. calc. for C_33_H_27_ClF_4_N_6_O_4_S (715.12): C 55.42, H 3.81, N 11.75. Found: C 55.47, H 3.73, N 11.86.

### 3.2. Antimicrobial Activity Evaluation

The antimicrobial activity of the compounds was tested on the Gram-positive strains (*Staphylococcus aureus* ATCC 25923, *Staphylococcus aureus* ATCC 6538, *Staphylococcus aureus* Microbank 14001, *Staphylococcus epidermidis* ATCC 12228, *Bacillus subtilis* ATCC 6633, *Bacillus cereus* ATCC 10876, *Micrococcus luteus* ATCC 10240), and on the Gram-negative strains (*Escherichia coli* ATCC 25922, *Klebsiella pneumoniae* ATCC 13883, *Proteus mirabilis* ATCC 12453, and *Pseudomonas aeruginosa* ATCC 9027). Ciprofloxacin and vancomycin were used as control antibacterial agents. Microbial suspensions with an optical density of 0.5 McFarland standard 150 × 10^6^ CFU/mL (CFU—colony forming units) were prepared in sterile 0.85% NaCl. All stock solutions of the tested compounds were dissolved in dimethyl sulfoxide (DMSO). The medium with DMSO in the final concentration and without the tested compounds served as a control—no microbial growth inhibition was observed. Preliminary antibacterial *in vitro* potency of the tested compounds was screened using an agar dilution method on the basis of the growth inhibition on a Mueller-Hinton agar to which the tested compounds in concentration of 1000 µg/mL were added. In the next assay *in vitro* antibacterial activity of the compounds with inhibitory effect was determined by a broth microdilution method. The 96-well microplates were used; 198 μL of Mueller–Hinton broth with a series of two-fold dilutions of the tested compound in the range of the final concentrations from 0.24–1000 μg/mL was inoculated with 2 μL of microbial suspension. After incubation (at 37 °C for 18 h), spectrophotometric measurements of optical density (OD_600_) of the bacterial cultures with the tested compounds were performed in order to determine MIC. OD_600_ of bacterial cultures in the medium without the tested compounds was used as a control. The activity was expressed as the minimal concentration of the compound that inhibits the visible growth of the bacteria (MIC, minimal inhibitory concentration). The MBC (minimal bactericidal concentration), defined as the lowest concentration of each compound that resulted in >99.9% reduction in CFU of the initial inoculum, was also assessed.

### 3.3. Cytotoxicity Assay

HEK-293 (human embryonic kidney) cells were obtained from the American Type Culture Collection (ATCC CRL-1573) and were grown in Minimal Essential Medium (MEM; Sigma-Aldrich, Saint Louis, MO, USA) supplemented with 10% fetal bovine serum (FBS; Sigma-Aldrich). 100 U/mL of penicillin and 100 μg/mL of streptomycin were added to the media. The cell cultures were incubated at 37 °C in a humidified atmosphere with 5% CO_2_. The investigated compounds were dissolved in dimethyl sulfoxide (50 mg/mL) and then diluted in cell culture media supplemented with 2% FBS. HEK-293 cells were placed into 96-well plastic plates (Nunc, Roskilde, Denmark) at a cell density of 3 × 10^5^ cells per well. After 24 h of incubation at 37 °C, the media were removed and cells treated with the derivatives, diluted in media at final concentrations of 2–500 μg/mL. Cell cultures were incubated at 37 °C for 72 h. The cytotoxicity was estimated using 3-(4,5-dimethylthiazol-2-yl)-2,5-diphenyl tetrazolium bromide (MTT) that is cleaved into a colored formazan product by metabolically active cells, according to the assay described by Takenouchi and Munekata [15]. The quantity of the formazan product was measured in an automatic plate reader. From the obtained results the EC50 (concentration of the substance which inhibits cells growth in 50% in proportion to the growth of control cells) values were calculated. The results were given as mean ± SD of three independent experiments.

### 3.4. Enzymatic Assays

The inhibitory activity of DNA gyrase and topoisomerase IV from *E. coli* was evaluated using a gyrase supercoling assay kit and a topoisomerase IV decatenation kit (both kits obtained from Inspiralis, Norwich, UK). Briefly, supercoiled pBR322 plasmid DNA (0.5 μg) was incubated with 1 U gyrase, in the dedicated supercoiling assay buffer supplied by the manufacturer, in the presence of varying concentrations of the test compounds. Reactions were carried out at 37 °C for 1 h and then terminated by the addition of an equal volume of 2× STOP Buffer (40% sucrose, 100 mM Tris Cl pH 7.5, 1 mM EDTA, and 0.5 mg/mL bromophenol blue) and chloroform/isoamyl alcohol. Samples were vortexed, centrifuged and run through a 15 cm 1% agarose gel in TAE buffer (40 mM Tris-acetate, 2 mM EDTA) for 3 h at 50 V. Gels were stained with ethidium bromide and visualized under UV light. The decatenation assay was performed using an *E. coli* topoisomerase IV decatenation kit (Inspiralis). Interlinked kDNA substrate (0.5 μg) was incubated with 1 U topoisomerase IV (Inspiralis), in the dedicated decatenation assay buffer supplied by the manufacturer, in the presence of varying concentrations of the test compounds. Reactions were carried out at 37 °C for 1 h and then terminated by the addition of equal volume of 2× STOP Buffer (40% sucrose, 100 mM Tris-Cl pH 7.5, 1 mM EDTA, and 0.5 mg/mL bromophenol blue) and chloroform/isoamyl alcohol. Samples were vortexed, centrifuged and run through a 15 cm 1% agarose gel in TAE buffer for 1.5 h at 80 V. Gels were stained with ethidium bromide and visualized under UV light. Concentrations of inhibitor that prevented 50% of the kinetoplast DNA from being converted into decatenated minicircles (IC_50_ values) were determined by plotting the results obtained from the densytometric analyses of the gel images using Quantity One software (BioRad).

## 4. Conclusions

We have examined several novel CPX-triazole hybrids against pathogenic and opportunistic bacteria. A number of these compounds displayed enhanced potency both against Gram-negative and Gram-positive bacteria, including MRSA strain. What is important, their antibacterial effect was achieved at completely non-toxic concentrations for human cells. Moreover, it has been shown that the increased activity is not caused by the increased affinity of the obtained compounds towards bacterial type II topoisomerases constituting the primary molecular targets for fluoroquinolones. The research has also proven that the presence of the disubstituted phenyl ring connected to the 1,2,4-triazole skeleton determines stronger antibacterial activity rather towards Gram-positive than Gram-negative bacteria.
